# Postural Differences in Speaking Versus Non-Speaking Children with Autism Spectrum Disorder

**DOI:** 10.3390/children12020145

**Published:** 2025-01-27

**Authors:** Marta Będziechowska-Czyżewska, Roksana Malak, Mateusz Romanowski, Mirosław Andrusiewicz, Włodzimierz Samborski, Ewa Baum

**Affiliations:** 1 Department and Clinic of Rheumatology, Rehabilitation and Internal Diseases, Poznan University of Medical Sciences, 28 Czerwca 1956 r. 135/147, 61-545 Poznań, Poland; wlodzimierz.samborski@ump.edu.pl; 2 Greaterpoland Physiotherapy Center, Bohaterów II Wojny Światowej, 60-386 Poznań, Poland; m.romanowski@wcf.com.pl; 3 Department of Cell Biology, Poznan University Medical Sciences, Rokietnicka 5D, 60-806 Poznań, Poland; andrus@ump.edu.pl; 4 Department of Social and Human Sciences, Poznan University of Medical Sciences, Rokietnicka 7, 60-806 Poznań, Poland; ebaum@ump.edu.pl

**Keywords:** autism spectrum disorder (ASD), body posture, communication disorders, range of motion, popliteal angle test, spinal curvatures

## Abstract

**Background/Objectives:** Autism spectrum disorder (ASD) is a heterogeneous condition with diverse symptoms influenced by factors like gender, severity and the involvement of family and therapists. While many risk factors that contribute to ASD development are known, the exact etiology remains unclear. The relationship between speech ability and postural/gait patterns in ASD has not been extensively studied. This study aimed to verify if the ability to speak can affect body posture and gait patterns. **Methods:** The study involved 28 boys aged 6–17. The postural assessment used the Adams test, Bunnell scoliometer, goniometer, and inclinometer to measure trunk rotation, joint range of motion, and spinal curvature. Trunk muscle strength was assessed via a flexion test measuring position maintenance time. This study compare body posture parameters in speaking and non-speaking children with Autism Spectrum Disorders. Moreover the parameters were compared to the general norms. **Results:** The study observed a tendency for speaking children to deviate more from normative body posture. They presented shoulder protraction more often, increased lumbar lordosis angle, and anterior pelvic tilt. Additionally, non-speaking children were more prone to toe-walking, which, according to other studies, is present in approximately 8–9% of all children with autism spectrum disorders. Both groups presented a decreased angle of dorsal flexion in the ankle joint. **Conclusions:** This study suggests that speaking children with ASD exhibit greater anterior-posterior postural deviations (increased lumbar lordosis, shoulder protraction, anterior pelvic tilt) than non-speaking children. ASD did not affect scoliosis or trunk rotation. Non-speaking children showed a higher incidence of toe-walking. However, the small sample size limits the generalizability of these findings.

## 1. Introduction

### 1.1. Characteristics of Autism Spectrum Disorders

Autism spectrum disorder is a neurodevelopmental disorder syndrome. It includes communication difficulties, sensory processing disorders, motor deficits, challenges in interpersonal interactions, and may involve stereotypical behaviors [1,2,3,4,5,6,7,8,9,10].

According to the World Health Organization (WHO), in 2012, 1.5−2.5% of the population suffered from ASD, while in 2017, 1 per 160 children were diagnosed with ASD [2]. Recently, it was estimated that 1.5% of children in developed countries have ASD [8]. This disorder affects boys more often than girls [8]

There are many theories about autism spectrum disorder’s etiology, but none have been confirmed. It was initially believed to be a psychiatric disease [11]. Later, theories about brain and cerebellum development and their abnormalities emerged. It has evolved into the concept of neurodiversity [1]. Current researchers lean towards the genetic hereditary theory [1,8,11]

Although ASD is complex, according to the Diagnostic and Statistical Manual of Mental Disorders classification) (DSM-V), the autism spectrum is divided into three levels, with level 1 being the mildest and level 3 being the most severe [1]. The DSM-V classification notes that ASD features may co-occur with other dysfunctions or disorders, such as fragile X syndrome [12], attention deficit hyperactivity disorder (ADHD), epilepsy, food selectivity, gastroenterological problems, sleep disorders, depression, and anxiety disorders [1,7,11]. Children who have older siblings, or spend more time with their families, or visit physicians more frequently are typically diagnosed earlier compared to sole children or those with younger siblings. Girls, children without speech delays, and members of national minorities also tend to receive later diagnoses due to difficulties in assessing symptoms that may be ambiguous or masked [5,9].

### 1.2. Body Posture of Children with Autism Spectrum Disorders

Children with ASD generally exhibit an incorrect arrangement of body segments relative to each other [12,13,14]. Researchers have observed scoliosis of the lumbar part of the spine, incorrect parameters of thoracic kyphosis, valgus knee, head protraction, asymmetric shoulders, and protruding shoulder blades [1]. Additionally, there are balance disorders, attempts to increase the base of support, and increased movement dynamics [1,4,5,10].

More significant sideways swings were noticed in children with the autism spectrum during tests on postural control on stabilometric platforms. These deviations may result from sensory stimulus processing disorders, motor cortex dysfunction, or overstimulation [5,6,7,9,10,12,13]. Swings are controlled by dorsiflexion and plantarflexion in the ankle joint and movement in the hip joint [6,14]. Children with ASD may control their position using hip mechanisms and joint movements to place body elements in space. This mechanism involves maintaining balance in a standing position through adduction and abduction in the hip joint [1,5,6,12,14,15]. While movements in the ankle joints help shape postural control, children with ASD may not fully utilize this mechanism if it is underdeveloped [1,5,6,13,14,15].

Although up to 95% of parent report sensory processing abnormalities in their children with ASD no clear research explains the relationship between sensory integration disorder and body posture [6]. There have been documented avoidance of certain sounds or textures, unusual smelling of objects, seeking out visual experiences of lights or movement. However the body posture has not been described yet.

Children with ASD demonstrate slower development of postural control mechanisms compared to their peers. It explains their greater “clumsiness” and frequent falls [4,6,9,10,13,14,15]. We have not found in any researches the relationship between body posture and ability to speech in children with ASD. There have been described many important features in children with ASD and posture.

Analyzing literature and our clinical experience, we have found valid to search differences in body posture between children with ASD with ability to speech and non-speaking children- moreover what the research allowed for compare to general norms of body posture parameters.

The study aimed to determine whether body posture parameters in children with the autism spectrum (ASD) differ from norms and whether speech or its absence in children with ASD visibly influences the parameters describing body posture.

## 2. Materials and Methods

### 2.1. Characteristics of the Study Group

The study involved 28 boys aged 6–17 treated at the Greaterpoland Physiotherapy Center in Poznań. The average age of the patients was 10 ± 3.3 years. All children were of Caucasian descent. The study group included only males with a confirmed diagnosis of autism spectrum disorder.

### 2.2. Characteristic of the Speaking and Non-Speaking Group

The group was divided into two subgroups based on the presence or absence of spoken language. The first group consisted of speaking children (n = 17), and the second group consisted of non-speaking children (n = 11). Only boys participated in the study because no girls were found who met the study’s criteria, i.e., a diagnosis of autism spectrum disorder and obtaining written consent from the child’s parents or legal guardians. The number of participants depends on the total of patients, their health condition on the examination day, their cooperation with researchers and obtaining written consent from the child’s parent or legal guard.

### 2.3. Including and Excluding Criteria

The study was conducted based on informed, written consent from parents or legal guardians. Approval for the research was obtained from the Institutional Review Board of the Poznan University of Medical Sciences. Additionally, the IRB was asked to state that the study did not bear the hallmarks of a medical experiment. A positive opinion was issued, permitting the research (resolution number 203/23). In addition, consent was obtained from the director of the Greaterpoland Physiotherapy Center to conduct research at the facility. The study was conducted in accordance with the Declaration of Helsinki (2013).

Two children were excluded from subsequent analyses due to the inability to obtain actual measurements due to the co-occurrence of additional diseases. Participants were excluded if they had comorbidities that hindered examination, infections, fractures, or were unwell on the measurement day.

### 2.4. Aim of the Study

The study aimed to determine whether body posture parameters in children with the autism spectrum (ASD) differ from norms and whether speech or its absence in children with ASD visibly influences the parameters describing body posture.

### 2.5. Methods

The study was cross-sectional. The data was collected at a single point in time. The researchers assessed the posture and speech abilities of the participants only once. There was no follow-up over time to observe changes. We observed and measured pre-existing characteristics (postural measurements and speech ability) without intervening or manipulating any variables. We observed and recorded the data as it naturally existed.

The Adams test and Bunnel scoliometer were used to determine the trunk rotation angle. The measurement consisted of bending over and checking the trunk rotation angle at four levels: proximal thoracic, main thoracic, lumbar, and pelvic [16].

The normal range is 3–4°, which does not require treatment. Scoliosis can be suspected when the trunk rotation angle is 5–7°; this result should be monitored, and possible progression should be checked. A result above 7° indicates the need for control and therapeutic activities [16,17,18].

The ranges of motion in the joints were measured using a goniometer placed on the joint axis. The stationary arm pointed to the center of the stationary bone, and the moving arm followed the center line of the moving part [19].

The physiological range of motion in the ankle joint is 10–20° dorsiflexion, which correlates with the proper length of the gastrocnemius and soleus muscles [16,19]. The range of motion of dorsiflexion in the ankle joint, measured in a sitting position with the leg straight at the knee joint, corresponds to the combined length of the gastrocnemius and soleus muscles without distinguishing between them [19]. The plantar flexion angle was measured in the same position, which should be 45–60° [20].

The popliteal angle was measured in the supine position with the lower limb bent at the hip, knee, and ankle joints to 90°. The angle was measured from the vertical to the maximum passive extension of the knee joint [21]. The range of motion for the popliteal angle should be under 30–40° [22].

The physiological varus knee occurs up to 2 years old, and the valgus knee occurs up to 6 years old [23].

The range of motion in the hip joint was measured in several positions. Extension was measured in the prone position, and internal and external rotation were measured in the same position. The extension involved passively lifting the lower limb with pelvic stabilization. The standard extension in this joint is 10–15° [20]. Rotations were checked when the lower limb was bent at the knee joint to an angle of 90°, the movable arm of the goniometer followed the center of the tibia, and the stationary arm pointed to the vertical. The usual range of internal and external rotations is 30–45°. Flexion was measured in the supine position and involved passively bending the lower limb and pulling it to the abdomen [16]. The normal flexion range is between 125–140° [20].

The spine curvatures in the sagittal plane were measured while standing in a relaxed, unforced position, with arms along the body and feet hip-width apart. An inclinometer was placed on the sacrum to check the angle of the sacrum, then zeroed to examine the relative angle and placed on the thoracolumbar junction to check the angle of lumbar lordosis. It was then zeroed on the thoracolumbar junction and placed on the cervical-thoracic junction to determine thoracic kyphosis [2,21,22]. The normal range of spinal curvatures should be 20–40° [23,24].

The assessment of the position of the head, shoulders, shoulder blades, waist angles, pelvis, knees, and feet were assessed in a relaxed standing position, with the lower limbs positioned hip-width apart and the eyes looking straight ahead [25].

The flexion test (bending test, V – sit test) consisted of testing the strength and endurance of the trunk muscles (rectus abdominis and the obliques abdominis muscles) It involved keeping the upper limbs straight and the knee and hip joints of the lower limbs bent at 90° while lying on the back. The time to maintain the position was measured in seconds [26,27,28,29,30,31]. 

### 2.6. Statistical Analyses

The study analyzed individual parameters in relation to groups and generally accepted standards. Statistical analysis was performed using Statistica version 13.3 software (Dell Corp, Tulsa, UK, USA).

The Shapiro-Wilk W test was used to assess the normality of the distribution of quantitative variables. The Student’s *t*-test was used for variables with a parametric distribution, and the Mann-Whitney *U* test was used for variables with a non-parametric distribution. The categorical data were compared using chi-square tests (Cochran’s adjusted).

The study’s sample size was restricted by the population of children diagnosed with autism spectrum disorder and meeting inclusion criteria at the collaborating clinic, thereby limiting the statistical power and generalizability of the results. The sample size was determined a posteriori following data collection. As the fractions of speaking to not-speaking children was 61% to 39% respectively, the statistical power was 0.73 for the group of 28 children.

The mean ± standard deviation (*M* ± *SD*) or median and lower-upper quartile range (*Me* [Q1–Q3]) were used to describe the study results. The coefficient of variation (CoV) was used to measure the variability (≤20%—low, ≤70%—moderate, ≤110%—high, and >110% very high variability). The level of statistical significance was assumed to be *p* < 0.05.

## 3. Results

The average age of the studied boys was 10 ± 3.3 years (*Me* = 9.5 [7–12] years, ranging from 6 to 17 years). Overall group coefficient of variation (CoV) by age indicated moderate variability (33%). In the speaking group, the average age was 10 ± 2.7 years (*Me* = 9 [6–12] years, range from 6 to 15). The CoV of the group in terms of age was 26%. In the non-speaking group, the average age was 10 ± 4 years (*Me* = 6 [6–12] years, range from 6 to 17). The CoV of this group was greater than that of the speaking group and amounted to 44%. There was no significant difference in the age of both groups (*p* = 0.088).

The average height of the studied boys was 1.46 ± 0.22 m (*Me* = 1.48 [1.37–1.63] m, range from 1.04 to 1.87 m). The CoV in height was low (15%). After division, the average height of the speaking group was 1.49 ± 0.171 m (*Me* = 1.48 [1.4–1.62] m, range 1.21 to 1.87 m). Group CoV in height was low (11%). In the group of non-speaking individuals, the average height was 1.41 ± 0.294 m (*Me* = 1.5 [1.05–1.65] m, range from 1.04 to 1.81 m). The CoV of this group in terms of height is greater and amounts to 20%. There was no significant difference in height between the groups (*p* = 0.410).

The average body weight of the boys was 41.44 ± 14.76 kg (*Me* = 42 [28–52] kg, ranging from 17 to 70 kg). The total CoV in body weight was moderate (35%). In the speaking group, the average body weight was 41.67 ± 13.43 kg (*Me* = 42 [30–45] kg, range 17–70 kg). The variability in this group was 32%. Non-speaking children’s average body weight was 41.09 ± 17.30 kg (*Me* = 40 [21–60] kg, range from 20 to 67). The variability in this group is greater and amounts to 42%. There was no significant difference in body weight between the groups (*p* = 0.921).

The average body mass index (BMI) of the examined children was 18.74 ± 2.84 kg/m^2^ (*Me* = 19.32 [16.94–20.30] kg/m^2^, range from 11.06 to 24.49 kg/m^2^). The CoV in the entire group was small and amounted to 15%. Among the speaking children, the average BMI was 18.06 ± 2.60 kg/m^2^ (*Me* = 19.17 [16.73–19.84] kg/m^2^, ranging from 11.06 to 20.54 kg/m^2^. The CoV in this group was also small (14%). In the group of non-speaking boys, the average BMI was 19.79 ± 3.00 kg/m^2^ (*Me* = 20.41 [17.78–22.04] kg/m^2^, ranging from 15.26 to 24.49 kg/m^2^). The CoV in this group was 15%. There was no significant difference in BMI observed between the groups (*p* = 0.264).

The average percentile of the study group was 45 ± 25.79 pth (*Me* = 44 [33.5–61.5] pth, range from 0 to 100). The CoV in the entire group was higher but still at an average and acceptable level (56%). Among the speaking children, the average percentile was 40 ± 23.20 pth (*Me* = 41 [34–47] pth, ranging from 0 pth to 100 pth. The CoV in the speaking group was 57%. Among non-speaking boys, the mean percentile is 53 ± 28.50 pth (*Me* = 61 [33–68] pth, range from 0 to 100). The CoV in this group was 53%. There was no significant difference in percentile between the groups (*p* = 0.179). Parameters of body posture in speaking versus non-speaking group are shown in Table 1.

When analyzing the thoracic kyphosis angle, no significant differences were observed between speaking and non-speaking boys (*p* = 0.327). However, non-speaking children had a slightly higher median thoracic kyphosis angle of *Me* = 36° [20–40]° compared to speaking children, *Me* = 33° [27–51]°. The boys were also divided depending on whether the kyphosis angle they presented was within the normal range of 20–40° or beyond it. There were no statistically significant differences between speaking and non-speaking children whose results were within the norm and those beyond it (*p* = 0.281).

Analysis of the lumbar lordosis angle showed that verbal boys tended to have a higher lumbar lordosis angle than non-verbal boys (*p* = 0.083). The average value of lumbar lordosis in the speaking group was *M* = 47 ± 18.6° and in the non-speaking group *M* = 36 ± 7°. However, no significant difference was found between the groups.

No statistically significant differences were found when comparing the speaking and non-speaking groups regarding the norm and non-normal lumbar lordosis angle (20–40°). There was, however, a clear tendency (*p* = 0.058) for speaking individuals to have a lumbar lordosis angle outside the norm, while non-speaking boys had a normal lumbar lordosis angle.

Regarding the angle of inclination of the sacrum, no statistically significant difference was observed between speaking and non-speaking boys (*p* = 0.229). However, speaking children showed a slightly higher median sacrum angle, *Me* = 27° [23–40]°, compared to non-speaking children, *Me* = 25° [17–30]°.

Boys were also grouped depending on whether the angle of the sacrum was within the norm (20–40°) or beyond it. There were no statistically significant differences between the groups (*p* = 0.421).

When analyzing the angles of rotation of the trunk in relation to speech in the proximal thoracic (*p* = 0.704), main thoracic (*p* = 0.669), lumbar (*p* = 0.684), and pelvic level (*p* = 0.244), no significant differences were found between speaking and non-speaking individuals.

A positive result indicates rotation to the right, while a negative result indicates rotation to the left. The average angle of trunk rotation in the proximal thoracic section for the speaking group was *M* = 0 ± 2.78°; in the non-speaking group, *M* = 1 ± 2.71°. In the main thoracic section, the mean rotation angle in the group of speaking boys was *M* = 0 ± 4.57°. In the group of non-speaking boys, *M* = (−1) ± 4.08 For the lumbar section, the average rotation angle was *M* = 0 ± 5.65° for the speaking group, and *M* = (−1) ± 3.62° for the non-speaking group. At the pelvic level, the distribution was not consistent with the parametric distribution. Therefore, the median for the speaking group could be determined to be *Me* = 0° [0–2]°, and for the non-speaking group, *Me* = 0° [(−1)–0]°.

Taking into account the division into normal parameters (up to 4° of rotation to the left or right), no significant differences or trends were observed in the speaking and non-speaking groups in the following sections: proximal thoracic (*p* > 0.999), main thoracic (*p* = 0.447), lumbar (*p* = 0.405), pelvic level (*p* = 0.362).

The range of ankle dorsiflexion differed significantly between the speaking and non-speaking groups. Non-speaking children showed a greater tendency to reduce this angle (*p* = 0.049). In the group of non-speakers the median was *Me* = 0° [(−2)–0]°, whereas in the speaking group it was *Me* = 0° [0–7.5]°.

When comparing the range of dorsiflexion movement to the normal range (10–15°) between the speaking and non-speaking groups, no significant differences were observed (*p* > 0.999). Most subjects (89%) had a range of ankle dorsiflexion movement below the norm.

There were no significant differences between speaking and non-speaking groups in plantar flexion (*p* = 0.210). However, the average range of plantar flexion in the speaking group was M = 51° ± 10.6°, and in the group of non-speaking boys, it was greater *M* = 57° ± 11.8°.

Compared to the normal plantar flexion range (45–60°), no statistically significant differences were observed between the speaking and non-speaking groups (*p* = 0.315).

No significant differences were observed between speaking and non-speaking boys regarding hip extension (*p* = 0.546). The medians in the groups did not differ significantly, with speaking children showing a lower median of *Me* = 8° [5–10] than non-speaking children *Me* = 10° [5–12.5]°.

No significant differences were observed between speaking and non-speaking children after categorizing parameters within the norm (10–15°) and beyond the norm (*p* = 0.139).

Similarly, in terms of hip flexion, no statistically significant difference was observed between speaking and non-speaking children (*p* = 0.54). However, speaking children had a higher median, *Me* = 140 [140–140]°, compared to non-speaking children, *Me* = 130 [120–140]°.

Compared to the normal hip flexion range (125–140°), no differences were observed between the groups (*p* = 0.652).

When analyzing rotational movements in the hip joint, no differences were observed between the groups regarding internal rotation (*p* = 0.276) or external rotation (*p* = 0.631). The average internal rotation movement was slightly higher in the speaking group, *M* = 45° ± 12.8°, compared to the non-speaking group, *M* = 39° ± 14.9°. Conversely, the average range of external rotation was higher in the non-speaking group, *M* = 41° ± 13.6°, compared to the speaking group, *M* = 39° ± 11.5°.

Comparing the measurements to the norm (30–45°) for internal rotation and external rotation, no significant differences were observed (*p* = 0.483 and *p* = 0.823, respectively).

Considering the popliteal angle, no differences were observed between groups (*p* = 0.622). The average popliteal angle was slightly higher in the non-speaking group (*M* = 46 ± 13°), compared to the speaking group (*M* = 44 ± 14.6°).

When dividing all participants’ measurements into those within the norm (<30–40°) and beyond the norm, no differences were observed between the groups (*p* > 0.999). However, it was noted that the entire group tended to have an increased angle value; 86% of respondents showed a reduced extension range in the knee joint.

There was a significant difference in the time achieved by children in the flexion test (*p* = 0.009). Speakers showed significantly longer posture time with their upper and lower limbs raised. The median in the speaking children group was higher, *Me* = 21 [15–35]s, compared to the non-speaking group, *Me* = 17 [10–24]s.

When analyzing the position of individual body elements concerning each other and their arrangement in space, no significant differences emerged in head position: head in protraction (*p* = 0.575) and normal head position (*p* = 0.575).

However, in the position of the shoulders, there was a tendency for speaking children to place their shoulders in protraction (*p* = 0.097). In contrast, non-speaking children tended to have symmetrical shoulder positioning (*p* = 0.094).

For other observed variants of shoulder positioning—the right shoulder was higher (*p* = 0.353), and the left shoulder higher (*p* = 0.619)—no significant differences were observed between the groups.

There was also no difference observed in the position of the shoulder blades: shoulder blades protruding (*p* = 0.388), right shoulder blade higher (*p* > 0.999), rotated shoulder blades (*p* = 0.505), and symmetrical shoulder blades (*p* = 0.699). None of the individuals examined had their left shoulder blade positioned higher than the right.

The presence or absence of speech did not affect the position of the waist triangles: the right waist triangle increased (*p* > 0.999), the left waist triangle increased (*p* = 0.668), waist triangles were symmetrical (*p* = 0.705)

In the pelvis position, it was found that there is a strong tendency for speaking children to position the pelvis in an anterior tilt (*p* = 0.058). Posterior pelvic tilt did not occur in any of the subjects. No differences were observed in the pelvic oblique position between the speaking and non-speaking groups (*p* > 0.999). However, there was a weak tendency for non-speaking children to place their pelvis in an intermediate position (*p* = 0.097)

After analyzing the knee alignment, no significant differences were observed between the groups in knee hyperextension (*p* > 0.999), valgus alignment (*p* = 0.799), and normal alignment (*p* = 0.699). Knee varus did not occur in any of the examined boys.

The following foot positions were observed: flat feet (*p* = 0.388), valgus feet (*p* = 0.887), feet in internal rotation (*p* > 0.999), feet in external rotation (*p* > 0.999), and feet in intermediate position (*p* = 0.976). No significant differences were observed between speaking and non-speaking individuals in these positions.

There is a tendency for non-speaking individuals to demonstrate toe walking more often compared to speaking individuals (*p* = 0.076).

After analyzing full-term or premature birth, it was concluded that prematurity did not influence the presence or absence of speech in children with the autism spectrum (*p* > 0.999).

## 4. Discussion

The relationship between body posture and speech in children with ASD was the aim of our research. Although these are some dissertations that showed a correlation between body posture and acoustic speech, during the analysis of current studies, no scientific publication was found that describes the exact body posture of children in the autism spectrum as a single group or divides them into speaking and non-speaking children. The book”Autism in Children: Clinical Knowledge” briefly mentions body posture, but but these are observations by the authors rather than conducted research [1]. We found that it could be an interesting subject because somatosensory and proprioceptions neurons of the body are located in the dorsal root spinal ganglia [31,32,33,34]. Without proprioceptive neurons, the human body would not be able to properly perform movements due to the lack of transmission of information about stimuli from inside the body and from the surrounding world to the muscles as effectors [34,35,36]. An example of the proprioceptive work of muscles is acoustic speech, which engages many muscles in the body [37,38].

Boys are diagnosed with autism spectrum disorder earlier and more frequently than girls, up to four times more often [6]. The presentation of autism differs between genders, complicating swift and accurate diagnosis in girls. This study focused exclusively on a homogeneous group of boys meeting the criteria for autism spectrum disorder.

The study revealed differences in body posture between speaking and non-speaking individuals diagnosed with autism spectrum disorder. Furthermore, deviations from generally accepted standards were observed across the study group.

Certain angles of spinal curvature in the sagittal plane were found to correlate with speech. A statistically significant difference was noted in lumbar lordosis, indicating that speakers tend to have an increased lumbar lordosis angle exceeding 40°.

Combined with the tendency of examined speaking children to position their pelvis in an anterior tilt and their shoulders in protraction, their posture can resemble a sway-back posture. This posture is characterized by protraction of the head and shoulders, an increased angle of lumbar lordosis, anterior pelvic tilt, a reduced or normal range of hip flexion, as well as a normal or hyperextended knee position and an increased range of plantar flexion in the ankle joint [2].

In the sway-back stance, hypoactive muscles include the middle and lower parts of the trapezius dorsi, serratus anterior, rhomboid major and minor, erector spinae in the lumbar part, internal abdominal obliques, iliacus, and gluteus maximus. Hyperactive muscles include the thoracic part of the erector spinae, rectus abdominis, external abdominal oblique, lumbar, sternocleidomastoid, scalene, upper part of the trapezius dorsi, pectoralis major and minor, and the posterior group of thigh muscles [2].

In numerous studies involving children with the autism spectrum, displacements of the center of gravity have been observed on stabilimeter platforms [1,5,6,7,9,10,13,15]. It has been demonstrated that an increase in the angle of lumbar lordosis affects the movement of the center of gravity during activities such as walking in the frontal plane. At the same time, in a standing position, deflections to the left and forward occur [1,6,13,15]. These disorders contribute to the general “clumsiness” reported by parents of children on the autism spectrum [4,5,6,13,14,15].

However, the posture of examined non-speaking children can be compared to a normal posture, where the head is in a neutral position, the shoulders are symmetrical, and the pelvis is in an intermediate position. A plumb line drawn from the ear should pass through the center of the shoulder joint, slightly posterior to the axis of the hip joint, in front of the knee joint, and end at the lateral malleolus [2].

The body posture of speaking and non-speaking children in the study group does not resemble prematurely born children exhibiting marked lumbar lordosis, shallow thoracic kyphosis, and a retroverted pelvis [27]. Considering that the majority (80%) of the examined children on the autism spectrum were born at term, it can be assumed that they would present a different body posture pattern.

The multifidus, transverse abdominis, diaphragm, pelvic floor, interspinous, intertransverse, trapezius lumborum, and extensors stabilize the spine and surrounding joints [2,28]. The basis for maintaining body posture is appropriate muscle tension [24,28].

Sensory integration disorders and hypersensitivity to stimuli may co-occur with autism spectrum disorder. One of the theories for the appearance of toe walking in this group is the disturbance in the processing of external stimuli. To avoid contact with unpleasant surfaces, individuals on the autism spectrum raise their heels [3,4]. It reduces the area of contact with the ground [3,4]. The study observed a more frequent occurrence of toe walking among non-speaking children, who were more likely to develop stereotypical behaviors, hypersensitivity to various types of stimuli, and fixations. Non-speaking boys in other researches are typically classified at the lower levels of the DSM-V classification, typically up to level 3, where there are significant difficulties with verbal communication [1]. These children do not speak or only use a few words, which, combined with rigid patterns and stereotypical behavior, makes it difficult for them to function in society [1]. Toe walking may occur when experiencing strong emotions or playing until age of 3 [4,5].

Due to the observed toe-walking pattern, non-speaking boys tended to have a smaller range of dorsiflexion motion than the speaking children. It may indicate a fixed position of the foot in plantar flexion, which can lead to shortening of the soleus and gastrocnemius muscles [3,4,19]. However, shortening muscle fibers in children on the autism spectrum does not cause foot positioning when walking, running, or standing [4,19]. Shortening is only a consequence of this habit [19]. Moreover, with great concentration on performing a given task, children can touch the ground with their heel and maintain this position for a short time [5]. It explains the heel-lifting mechanism as a defense against unwanted stimuli, which, when focused on an action, the child’s brain does not register as unbearable and irritating [3,4,5,19]. Some authors compare the gait pattern of non-speaking children to Parkinsonian or ataxic gait, in which the range of motion in the ankle joint is reduced, and step lengths vary [1,12]. Walking on toes affects balance and increases the likelihood of falling [5].

Regardless of the presence of speech, 89% of the subjects had an ankle dorsiflexion range below the norm of 10–15°, with most subjects having a range close to 0°. In most subjects, the reduced dorsiflexion range was not influenced by toe walking, as this was observed in only 25% of all subjects, without distinguishing between speaking and non-speaking children. This condition could be due to the lack of development of the ankle joint mechanism and the necessary range of motion to control body movements. In children on the autism spectrum, these mechanisms do not develop properly, and dorsiflexion remains at a lower level. They only use the hip mechanism they developed earlier to maintain a stable standing position by moving their hip joints [1,5,6,12,14,15].

Other studies have also shown that children on the autism spectrum who walked on their toes also had a reduced range of dorsiflexion motion [4,12,19]. In the case of children on the autism spectrum, cutting the Achilles tendon and plastering are not effective methods of treating toe walking. Much better results are achieved by combining physiotherapy with cooperation from parents, educators, and speech therapists in a comprehensive therapy approach [3,19].

Rehabilitation of children on the autism spectrum should be based on tasks that improve static and dynamic balance, influence the external senses, and enhance the understanding of one’s own body in space and motor coordination [7]. The stimuli reaching the child from the outside world during therapy are selected to stimulate appropriate receptors in the skin, tendons, muscles, and joints to elicit an adequate response from the effectors [28]. The pathway can both connect directly to alpha motor neurons or interneurons located at the same segment of the spinal cord or can travel upward or downward and establish connections with either interneurons or alpha motor neurons at adjacent spinal cord levels, but it can also ascend to form the dorsal column tract and synapse with neurons in the dorsal column nuclei within the brainstem and later to the neocortex [32]. Motor neuron stimulating the muscles to respond appropriately to the situation [28].

Despite the increase in the norm of the popliteal angle from 10° to 30°, the entire study group tended to reduce the range of motion. The curvature of the spine influences the position of the knees. The angle of lumbar lordosis affects how the knees are positioned as a mechanism to compensate for a decreased or increased angle of lumbar lordosis [24]. Similarly, the weakening of the gluteal muscles affects the position of the knee joints through the fascia of the back of the thigh and the lumbar part of the spine, to which the gluteal muscles connect [23].

In a flexion test, the strength of the flexors was checked, and better results were achieved by children who could communicate verbally. However, this result was not necessarily solely due to the weakening of the abdominal muscles. A shorter time in maintaining a position with raised upper and lower limbs could result from not understanding the command, reluctance to adopt a specific position, confusion caused by an additional person in the room where therapy usually took place, or focusing on something meaningful to the child. This test also examined proprioception due to a forced flexion position in which the posterior cords of the spinal cord were stimulated. The bodies of proprioceptive neurons are located in the dorsal spinal ganglia [29,30]. Without proprioceptive neurons, the human body would not be able to properly perform movements due to the lack of transmission of information about stimuli from inside the body and from the surrounding world to the muscles as effectors [29,30].

The human body functions as a unified whole; therefore, changing one parameter in body posture affects other elements, impacting both the ability and quality of locomotion [31,33]. It is important to notice, that individuals frequently misperceive their own body posture. This inaccuracy is evident in autism spectrum disorder, where postural differences and deviations from typical posture are common, especially among those with communication challenges. Neurodevelopmental variations in ASD directly affect sensory processing and motor control, consequently impacting posture. The observed differences in postural patterns between speaking and non-speaking autistic children highlight the significant neurological influence. Similar misperceptions of body posture are also observed in conditions like scoliosis, where individuals may inaccurately identify the location, severity, and orientation of their spinal curves. In scoliosis, this misperception can be attributed, in part, to proprioceptive dysfunction, further emphasizing the role of neurological factors in shaping both body awareness and postural perception [39].

Sensory processing disorders, which frequently occur in children on the autism spectrum, affect everyday functioning [1,3,4,5,7,19]. Considering the shorter time achieved by non-speaking children in the flexion test, which evaluates muscle strength and proprioception, and the more frequent occurrence of toe walking in this group, it is evident that non-speaking individuals have more significant difficulties in processing and reacting to internal and external stimuli. The toe-walking pattern helps reduce the contact area of the foot with the ground [3,4], and reluctance or quick abandonment of the flexion position indicates an escape from an uncomfortable, not fully controlled situation [28,29].

### Limitations of the Study

Primary limitation of this study is the small sample size, which resulted in a limited number of statistically significant findings and may affect the generalizability of the results. Sample size was depended of number of patients in Greater Poland Physiotherapy Center, their health condition, cooperation and consent of their parents or legal guardians. This study is cross-sectional study, we collect data only once, therefore it is more difficult to predict health condition in examination day and presence and absence of participants. Future research with a larger and more diverse sample is needed to confirm these preliminary observations.

## 5. Conclusions

It can be concluded that non-speaking individuals with autism spectrum disorders may present smaller spine deviations in the anterior-posterior plane, and the angles of spine curvature in the sagittal plane will be lower than those who use speech. However, individuals with verbal abilities may present more significant deviations in posture in the anterior-posterior plane. An increased lumbar lordosis angle, shoulder protraction, and an anterior pelvis tilt may be more common in this group. The autism spectrum does not influence the occurrence of scoliosis among children, and there was no tendency to increase the angle of trunk rotation at any of the levels tested. Due to more significant disorders in other areas, non-speaking individuals often present an abnormal gait pattern, such as walking on their toes.

## Figures and Tables

**Table 1:** Parameters of body posture in speaking versus non-speaking group.

Parameter Observed	*p*-Value
Thoracic kyphosis angle	0.327
Lumbar lordosis angle	0.083
Normal vs. beyond the norm lumbar lordosis	0.058
The angle of the sacrum	0.229
Normal vs. beyond the norm angle of the sacrum	0.421
Trunk rotation angle in proximal thoracic segment	0.704
Trunk rotation angle in main thoracic segment	0.669
Trunk rotation angle in lumbar segment	0.684
Trunk rotation angle on pelvic	0.244
Normal vs. beyond the norm trunk rotation angle in proximal thoracic segment	>0.999
Normal vs. beyond the norm trunk rotation angle in main thoracic segment	0.447
Normal vs. beyond the norm trunk rotation angle in lumbar segment	0.405
Normal vs. beyond the norm trunk rotation angle on pelvic	0.362
Range of dorsiflexion in the ankle joint	**0.049**
Normal vs. beyond the norm range of dorsiflexion in the ankle joint	0.999
Range of plantarflexion in the ankle joint	0.210
Normal vs. beyond the norm range of plantarflexion in the ankle joint	0.315
Range of extension in hip joint	0.546
Normal vs. beyond the norm range of extension in hip joint	0.139
Range of flexion in hip joint	0.547
Normal vs. beyond the norm range of flexion in hip joint	0.652
Range of internal rotation in hip joint	0.276
Normal vs. beyond the norm range of internal rotation in hip joint	0.483
Range of external rotation in hip joint	0.631
Normal vs. beyond the norm range of external rotation in hip joint	0.823
Range of popliteal angle	0.622
Normal vs. beyond the norm of popliteal angle	>0.999
Time obtained in the bending test	**0.009**
Head protraction	0.575
Normal position of head	0.575
Shoulders protraction	0.097
Symmetrical shoulder position	0.094
Left shoulder higher than right	0.619
Right shoulder higher than left	0.353
Shoulder blades sticking out	0.388
Right shoulder blades higher than left	>0.999
Rotated shoulder blades	0.505
Symmetrical shoulder blades	0.699
Increased right waist triangle	0.999
Increased left waist triangle	0.668
Symmetrical waist triangles	0.705
Anterior pelvic tilt	0.058
Pelvic oblique position	>0.999
Intermediate pelvic position	0.097
Knee hyperextension	>0.999
Valgus knee	0.799
Normal knee position	0.699
Flat foot	0.388
Valgus foot	0.887
Internal rotation of the foot	0.999
External rotation of the foot	0.999
Intermediate foot position	0.976
Toe walking	0.076
Prematurity	0.999

*p*-values < 0.05 are indicated in bold.

## Data Availability

The anonymized dataset used and/or analyzed during the current study is available from the corresponding authors on reasonable request.
